# Scutellarin Alleviates Neuronal Apoptosis in Ischemic Stroke via Activation of the PI3K/AKT Signaling Pathway

**DOI:** 10.3390/ijms26052175

**Published:** 2025-02-28

**Authors:** Zhaoda Duan, Yingqi Peng, Dongyao Xu, Yujia Yang, Yuke Wu, Chunyun Wu, Shan Yan, Li Yang

**Affiliations:** 1 School of Basic Medicine, Kunming Medical University, Kunming 650500, China; 20241963@kmmu.edu.cn (Z.D.); 18725295379@vip.163.com (Y.P.); xdy522469@163.com (D.X.); yujiayang1172@163.com (Y.Y.); 14787845493@163.com (Y.W.); wuchunyunkm@163.com (C.W.); 2 Institute of Biomedical Engineering, Kunming Medical Univesity, Kunming 650500, China

**Keywords:** ischemic stroke, scutellarin, neuronal apoptosis, PI3K/AKT pathway, network pharmacology, molecular docking

## Abstract

Among all stroke types, ischemic stroke (IS) occurs most frequently, resulting in neuronal death and tissue injury within both the central infarct region and surrounding areas. This study explored the neuroprotective mechanisms of scutellarin, a flavonoid compound, through an integrated strategy that merged in silico analyses (including network pharmacology and molecular docking simulations) with both in vitro and in vivo experimental verification. We identified 1887 IS-related targets and 129 scutellarin targets, with 23 overlapping targets. PPI network analysis revealed five core targets, and molecular docking demonstrated strong binding affinities between scutellarin and these targets. Bioinformatic analyses, including GO functional annotation and KEGG pathway mapping, indicated that the PI3K/AKT cascade represents the primary signaling mechanism. An in vitro experimental system was developed using PC12 cells under oxygen-glucose deprivation conditions to investigate how scutellarin regulates neuronal cell death via the PI3K/AKT pathway. Western blot quantification demonstrated that treatment with scutellarin enhanced the expression of p-PI3K, p-AKT, and Bcl-2 proteins, while simultaneously reducing levels of apoptotic markers Bax and cleaved caspase-3. Furthermore, pharmacological intervention with the selective PI3K inhibitor LY294002 attenuated these molecular alterations, resulting in diminished expression of p-PI3K, p-AKT, and Bcl-2, accompanied by elevated levels of Bax and cleaved caspase-3. In a rat model of middle cerebral artery occlusion, scutellarin administration demonstrated comparable neuroprotective effects, maintaining neuronal survival and modulating apoptotic protein expression via PI3K/AKT pathway activation. Collectively, this study demonstrates the therapeutic potential of scutellarin in cerebral ischemia through PI3K/AKT pathway modulation, suggesting its possible application in treating ischemic disorders.

## 1. Introduction

Cerebrovascular accidents, commonly known as strokes, represent severe medical emergencies resulting from compromised blood vessel integrity or blockage in the brain, leading to substantial rates of death and permanent disability. The global burden of this condition shows a concerning upward trend each year, with developing nations experiencing a disproportionate increase in case numbers [1]. Among stroke variants, ischemic stroke (IS) represents the predominant form, constituting roughly four-fifths of all reported cases. IS pathophysiology encompasses multiple intricate processes, including metabolic disruption, glutamate-mediated excitotoxicity, inflammation, oxidative damage, and neural death [2,3]. Current therapeutic strategies primarily focus on reestablishing cerebral perfusion in the affected brain regions. Nevertheless, the effectiveness of reperfusion therapy faces limitations due to strict time constraints and potential complications associated with restored blood flow (I/R injury). Intravenous rtPA thrombolysis, despite being the sole FDA-sanctioned treatment, is constrained by limited timing requirements and potential adverse effects, including cerebral bleeding and edematous complications [4]. Therefore, the urgent development of drugs with multi-faceted neuroprotective effects is of paramount importance for preventing the disease and improving patient outcomes.

The unique therapeutic approach of TCM employs diverse active compounds targeting multiple biological pathways, demonstrating distinctive advantages in stroke management and prevention [5]. Research has extensively documented the effectiveness of TCM interventions against stroke, ranging from individual botanical agents to sophisticated herbal combinations and purified active constituents [6,7,8]. Yunnan Province dominates the national production of *Erigeron breviscapus*, a widely utilized medicinal plant, contributing to nearly all domestic supply. The bioactive component scutellarin, structurally characterized as 4,5,6-trihydroxyflavone-7-glucuronide, represents a principal flavonoid glycoside derived from *Erigeron breviscapus* [9]. In traditional Yi ethnic medicine of Southwestern China, the whole plant has been traditionally employed for the management of stroke-induced paralysis and rheumatic arthralgia [10]. It has been found that scutellarin, due to its high clinical efficacy and low toxicity, is widely used in clinical treatment. In IS, the neuroprotective mechanisms of scutellarin are predominantly attributed to its capacity to suppress neuronal apoptosis, modulate inflammatory cascades, and exert potent antioxidant effects. Research indicates that ischemic brain regions comprise two distinct zones: a permanently damaged central core and an encircling area of compromised tissue known as the penumbra. The infarct core consists of dead or dying tissue and is located at the center of the infarct area. Peripheral to the infarct core lies a region characterized by moderate hypoperfusion, termed the ischemic penumbra or peri-infarct zone [11]. The rapid depletion of oxygen and glucose reserves, coupled with ATP insufficiency, widespread cellular depolarization, excitotoxic cascades, and consequent tissue infarction in the core region following IS, renders immediate neuronal salvage virtually impossible. In contrast, the gradual progression of tissue deterioration within the ischemic penumbra creates a critical “therapeutic window,” affording an opportunity for cellular preservation and containment of infarct expansion [12,13]. Salvaging the potentially viable tissue within the penumbral zone represents a crucial therapeutic strategy, as successful intervention in this region can limit brain swelling and substantially improve patient outcomes post-stroke [14,15]. Accumulated evidence indicates that penumbral neurons undergo programmed cell death following cerebral ischemia, while maintaining cellular viability despite electrical dysfunction, as widely acknowledged in the scientific community [16,17]. Therefore, studying the potential mechanisms of neuronal apoptosis after IS and developing effective therapeutic strategies is of great significance for preventing further brain damage in IS patients and improving their prognosis.

Based on this, this study used online bioinformatics analysis to screen the potential core target network of scutellarin in relation to IS. Our research combined in vivo rat MCAO models with in vitro PC12 cell studies under oxygen-glucose deprivation to investigate scutellarin’s neuroprotective mechanisms, aiming to inform future therapeutic strategies and drug development efforts.

## 2. Results

### 2.1. Screening of Differentially Expressed Gene Targets in IS

**Figure 1:**
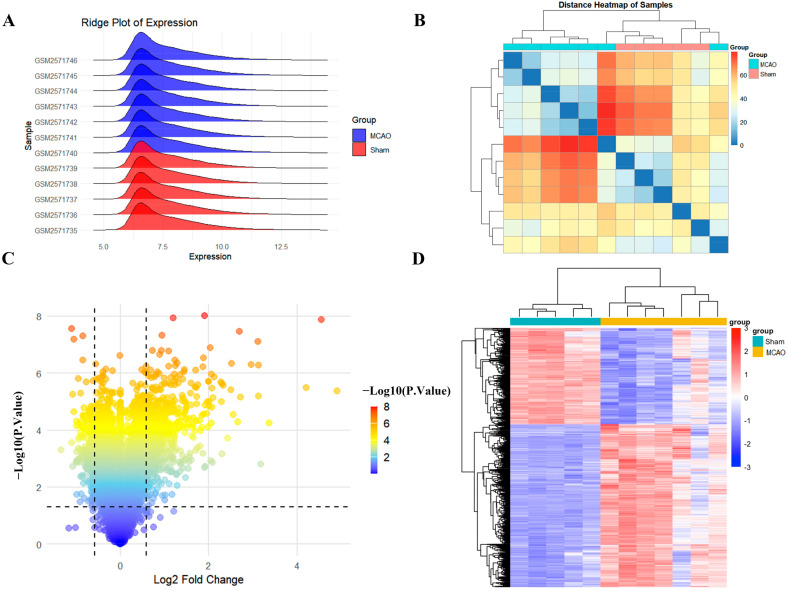
Differential expression analysis between MCAO and sham samples: (**A**) Distribution of gene expression in the samples. (**B**) Distance heatmap showing clustering relationships among the samples. (**C**,**D**) Volcano plot and heatmap illustrating differentially expressed genes.

The GEO microarray expression dataset GSE97537 consists of seven MCAO samples and five sham samples. The distribution and clustering of gene target expression between the MCAO and sham groups revealed significant differences in overall gene expression patterns (Figure 1A,B). Transcriptome analysis revealed 1887 differentially regulated genes, with 1185 showing increased expression and 702 exhibiting decreased expression levels (Figure 1C). The hierarchical clustering analysis of the 100 most significantly altered transcripts is presented as a heatmap visualization in Figure 1D. The observed expression patterns demonstrated high intragroup consistency while maintaining clear intergroup distinctions, establishing a robust foundation for subsequent molecular analyses.

### 2.2. Screening of Intersection Gene Targets Between Scutellarin and IS

**Figure 2:**
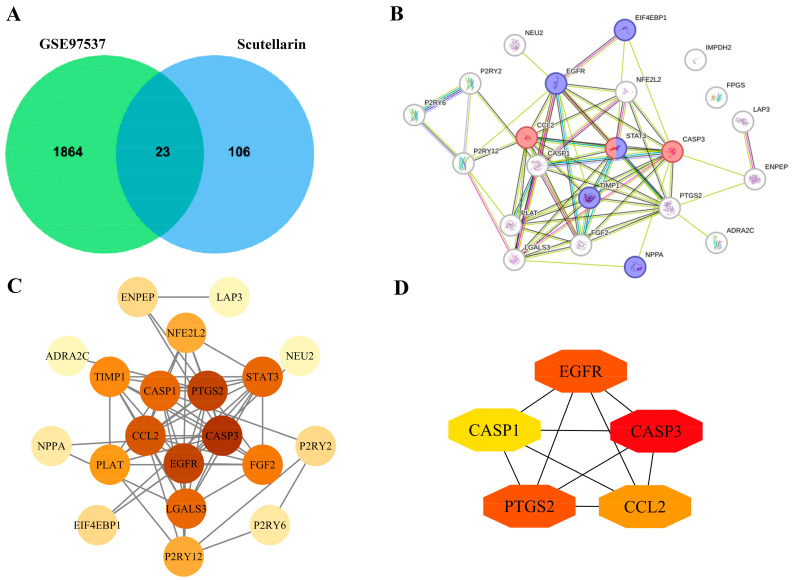
Screening of intersection gene targets: (**A**) Target intersection analysis between scutellarin and cerebral ischemia using Venn diagram. (**B**) STRING-based protein–protein interaction network construction. (**C**) Topological visualization of interaction networks. (**D**) Hierarchical representation of five key molecular targets based on degree centrality.

A total of 129 gene targets related to scutellarin in the treatment of IS were identified from database searches. A Venn diagram revealed 23 overlapping targets (Figure 2A). Network analysis of the 23 common targets via STRING platform generated an interaction map comprising 20 nodes with 65 distinct functional connections. Cytoscape 3.7.1 software enabled visualization of the protein–protein interaction network, where color intensity corresponded to correlation strength (Figure 2B,C). Topological analysis based on degree centrality metrics identified five pivotal molecular targets (Figure 2D, Table 1).

### 2.3. Go Function and KEGG Pathway Annotation Analysis

**Figure 3:**
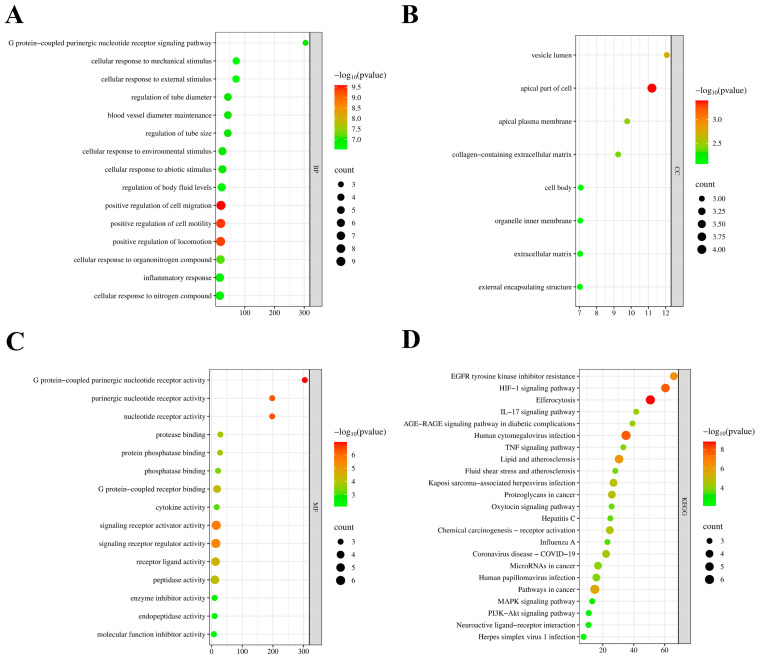
Functional annotation analysis: (**A**) Enriched biological processes visualization. (**B**) Cellular component distribution analysis. (**C**) Molecular function enrichment patterns. (**D**) KEGG pathway enrichment landscape.

Comprehensive functional characterization of the 23 identified overlapping targets was conducted through Gene Ontology enrichment and KEGG pathway mapping analyses. GO analysis identified 232 enriched terms categorized into biological processes (209), cellular components (8), and molecular functions (15), as illustrated in Figure 3A–C. Within the biological process category, predominant enrichment was observed in pathways regulating cellular movement, including migration, motility, and locomotion mechanisms. Analysis of cellular components revealed significant associations with apical cellular structures, plasma membrane regions, and vesicular compartments. Molecular function analysis revealed significant enrichment in purinergic signaling pathways, specifically involving G protein-coupled nucleotide receptor activities.

KEGG pathway mapping demonstrated significant enrichment in multiple signaling cascades, with prominent representation of PI3K-AKT signaling, cellular clearance mechanisms, hypoxic responses, growth factor signaling, and inflammatory pathways (Figure 3D). A bubble plot was generated to display the functional annotation findings.

### 2.4. Molecular Docking of Core Targets with Scutellarin

**Figure 4:**
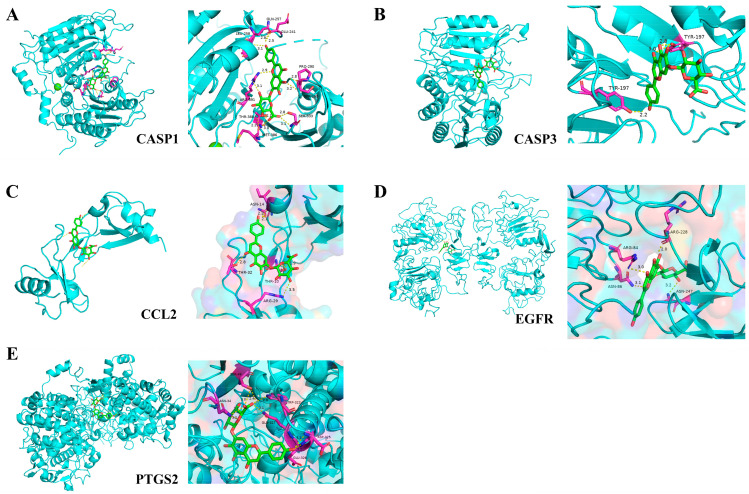
Molecular docking visualization of scutellarin with core targets: (**A**) Visualization of scutellarin docking with the CASP1 molecule. (**B**) Visualization of scutellarin docking with the CASP3 molecule. (**C**) Visualization of scutellarin docking with the CCL2 molecule. (**D**) Visualization of scutellarin docking with the EGFR molecule. (**E**) Visualization of scutellarin docking with the PTGS2 molecule.

Molecular docking analysis (Figure 4, Table 2) revealed strong hydrogen bonding interactions between scutellarin and CASP1 amino acid residues, exhibiting binding energy of −12.7 kcal/mol. Similar intermolecular interactions were observed with other key targets: CASP3 (−7.9 kcal/mol), CCL2 (−7.4 kcal/mol), EGFR (−9.0 kcal/mol), and PTGS2 (−9.9 kcal/mol), each demonstrating specific hydrogen bonding patterns. The negative binding energy values observed across all interactions suggest thermodynamically favorable spontaneous complex formation between scutellarin and its target proteins.

### 2.5. Evaluation of Scutellarin’s Neuroprotective Effects in MCAO-Induced Cortical Ischemia

**Figure 5:**
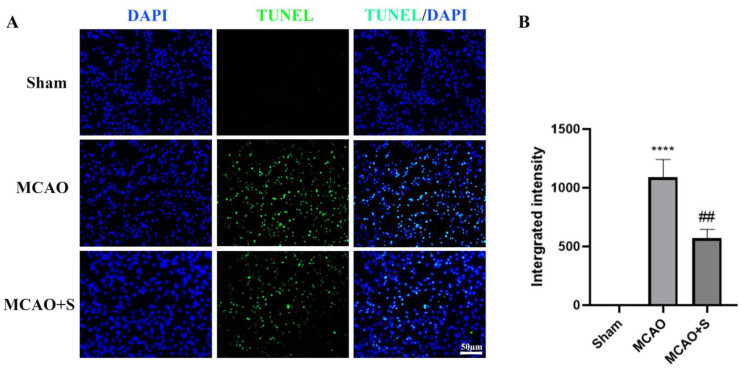
Scutellarin alleviates neuronal apoptosis in the ischemic cortex of MCAO rats at three days: Bar = 50 μm, *n* = 3. (**A**) TUNEL immunofluorescence images; (**B**) Quantitative analysis. **** *p* < 0.001 vs. Sham group; ## *p* < 0.01 vs. MCAO group.

Cortical tissue examination in sham-operated subjects showed no TUNEL-positive cells, indicating absence of apoptosis. MCAO induction resulted in a substantial elevation in TUNEL-positive neurons (visualized in green fluorescence) within the ischemic cortical region, relative to sham controls. In contrast, scutellarin administration significantly attenuated the MCAO-induced increase in apoptotic cell population, as evidenced by reduced TUNEL-positive signals in the treatment group (Figure 5). The results establish scutellarin’s neuroprotective efficacy against ischemia-triggered cortical cell death in the experimental MCAO model.

### 2.6. Assessment of Nissl Body Alterations in MCAO Rat Cortical Tissue Following 3-Day Scutellarin Administration

**Figure 6:**
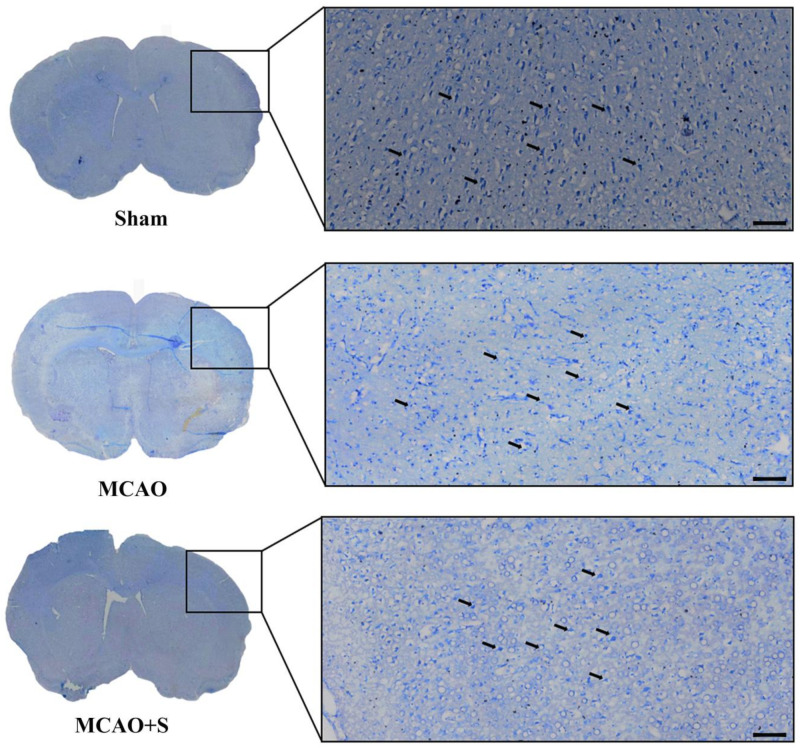
Effects of scutellarin on Nissl staining in the ischemic cortex of MCAO rats (×100).

Microscopic examination of Nissl-stained sections revealed well-preserved cellular architecture in the sham-operated group, characterized by distinct cellular boundaries and prominent Nissl substances, along with clearly observable nuclear components. The MCAO-induced injury resulted in diminished Nissl substance density, expanded extracellular gaps, and deteriorated cellular membrane definition relative to sham controls. In contrast, administration of scutellarin demonstrated therapeutic efficacy, as evidenced by enhanced Nissl substance preservation and attenuated cellular deterioration compared with untreated MCAO specimens (Figure 6). The findings demonstrate scutellarin’s protective effects against ischemia-induced neuronal deterioration in the MCAO rat cortex.

### 2.7. Effects of Scutellarin on the Phosphorylation Levels of PI3K and AKT in the Ischemic Cortex of MCAO Rats at 3 Days

**Figure 7:**
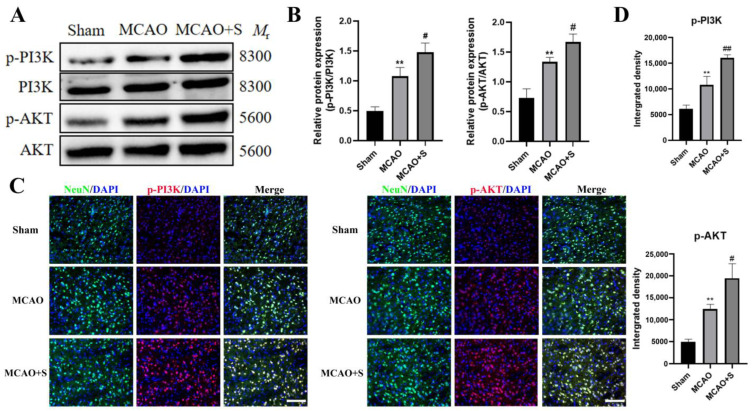
PI3K and AKT phosphorylation analysis in MCAO rat ischemic cortex following scutellarin treatment at day 3: (**A**,**C**) Representative Western blots and immunofluorescence micrographs; (**B**,**D**) Statistical analyses. Green fluorescence indicates NeuN-positive neurons, blue represents DAPI-labeled nuclei, and red shows Cy3-tagged proteins. Statistical significance: ** *p* < 0.01 vs. Sham; # *p* < 0.05, ## *p* < 0.01 vs. MCAO; Scale bar: 50 μm, *n* = 5.

Protein analysis via Western blotting demonstrated significant upregulation of phosphorylated PI3K and AKT expression in MCAO-subjected tissue compared to sham controls (*p* < 0.05). Administration of scutellarin further augmented the phosphorylation states of PI3K and AKT compared to untreated MCAO samples (*p* < 0.05, Figure 7A,B).

Fluorescence microscopy analysis revealed heightened immunoreactivity for phosphorylated PI3K and AKT, accompanied by elevated positive cell counts in MCAO specimens relative to sham preparations (*p* < 0.05). Post-scutellarin administration demonstrated further amplification of both immunofluorescence signals and phosphorylation-positive cellular populations compared to untreated MCAO tissue (*p* < 0.05, Figure 7C,D). These findings suggest scutellarin potentiates PI3K/AKT pathway activation through increased phosphorylation in this experimental paradigm.

### 2.8. Effects of Scutellarin on the Expression of Apoptosis-Related Proteins in the Ischemic Cortex of MCAO Rats at 3 Days

**Figure 8:**
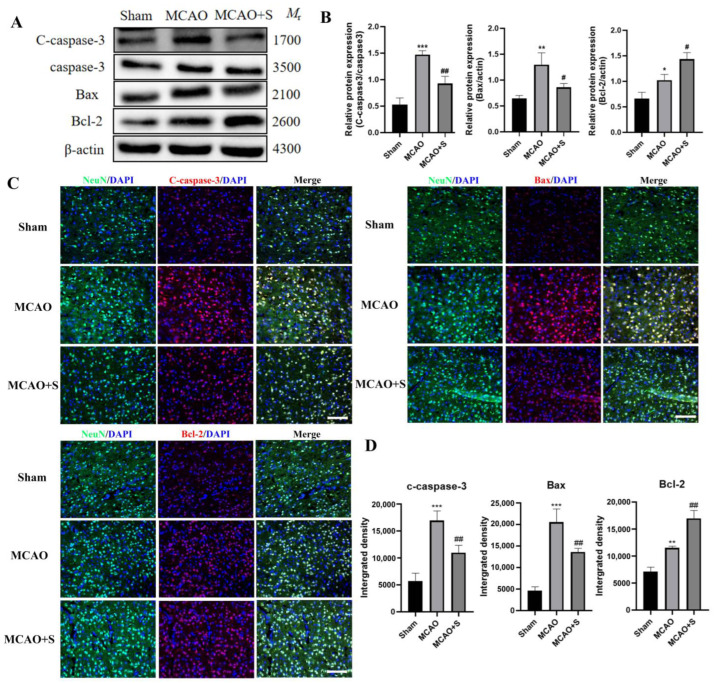
Apoptotic protein expression analysis in MCAO rat ischemic cortex after 3-day scutellarin treatment: (**A**,**C**) Western blot bands and immunofluorescence microscopy; (**B**,**D**) Quantitative data analysis. Neurons (NeuN, green), nuclei (DAPI, blue), target proteins (Cy3, red). Statistical significance: * *p* < 0.05, ** *p* < 0.01, *** *p* < 0.001 vs. Sham; # *p* < 0.05, ## *p* < 0.01 vs. MCAO; Scale = 50 μm, *n* = 5.

Protein analysis revealed marked elevation of both pro-apoptotic markers (Bax and activated caspase-3) and anti-apoptotic Bcl-2 protein levels in MCAO-subjected tissue versus sham controls (*p* < 0.05). Scutellarin administration resulted in dual regulatory effects: suppression of Bax and activated caspase-3 expression while enhancing Bcl-2 levels relative to untreated MCAO specimens (*p* < 0.05, Figure 8A,B).

Immunofluorescence analysis demonstrated intensified signal strength and elevated immunoreactive cell populations for all examined apoptotic markers in MCAO tissue compared to sham preparations (*p* < 0.05). Treatment with scutellarin induced differential regulation of apoptotic proteins, diminishing both the intensity and frequency of Bax and activated caspase-3 immunoreactivity while potentiating Bcl-2 expression parameters (*p* < 0.05, Figure 8C,D). The experimental data demonstrate scutellarin’s capacity to orchestrate reciprocal regulation of the apoptotic machinery, simultaneously suppressing death-promoting factors while enhancing survival signals.

### 2.9. Modulatory Effects of Scutellarin on the Transcriptional Regulation of Pathway-Enriched Genes in PC12 Cellular Model

**Figure 9:**
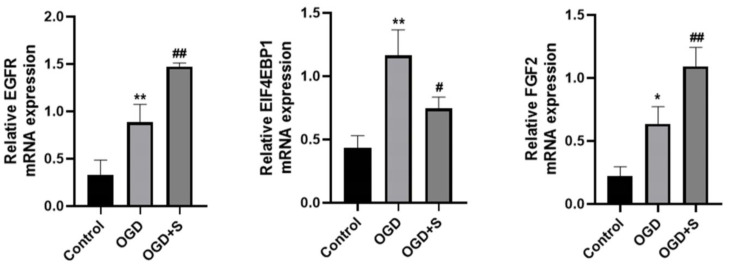
Effect of scutellarin on the mRNA expression of pathway-enriched genes in PC12 cells. Statistical significance: * *p* < 0.05, ** *p* < 0.01 vs. Control group; # *p* < 0.05, ## *p* < 0.01 vs. OGD group; *n* = 5.

Quantitative RT-PCR analysis revealed that oxygen-glucose deprivation induced significant elevations in the transcriptional levels of EGFR, EIF4EBP1, and FGF2 compared to the control conditions. Following scutellarin administration (OGD + S), a significant augmentation in EGFR and FGF2 transcription was observed, concurrent with a marked reduction in EIF4EBP1 expression relative to the OGD group (*p* < 0.05, Figure 9). These findings suggest that scutellarin may regulate neuronal apoptosis after IS by upregulating EGFR and FGF2 while downregulating EIF4EBP1 mRNA expression.

### 2.10. Effect of Scutellarin Combined with the PI3K Inhibitor LY294002 on PI3K, AKT Phosphorylation Levels, and Apoptosis-Related Protein Expression in PC12 Cells

**Figure 10:**
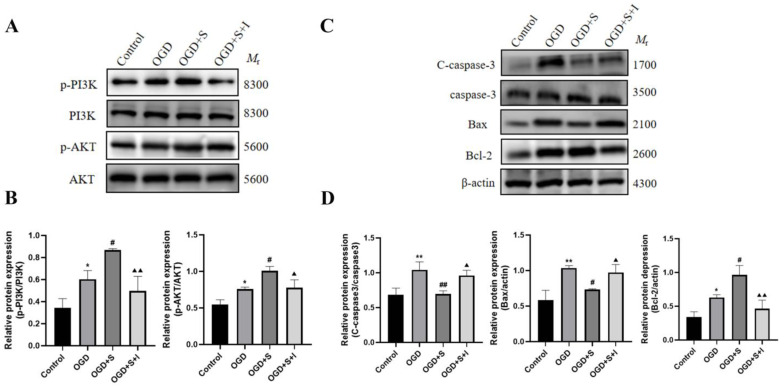
Impact of PI3K inhibition on scutellarin-mediated protein regulation in PC12 cells: (**A**,**C**) Western blot bands; (**B**,**D**) Quantitative data analysis. Statistical significance: * *p* < 0.05, ** *p* < 0.01 vs. Control; # *p* < 0.05, ## *p* < 0.01 vs. OGD; ^▲^ *p* < 0.05, ^▲▲^ *p* < 0.01 vs. OGD+S; *n* = 5.

The role of PI3K/AKT signaling in scutellarin-mediated neuroprotection was evaluated using the specific PI3K inhibitor LY294002. Scutellarin administration increased p-PI3K, p-AKT, and Bcl-2 levels while decreasing Bax and cleaved caspase-3 expression compared to OGD controls, as revealed by Western blotting. The addition of LY294002 (OGD + S + I) significantly attenuated scutellarin-induced upregulation of p-PI3K, p-AKT, and Bcl-2, while concurrently potentiating the expression of pro-apoptotic markers Bax and cleaved caspase-3 (*p* < 0.05, Figure 10). These findings demonstrate that scutellarin’s neuroprotective effects operate through PI3K/AKT pathway activation to suppress apoptotic processes.

## 3. Discussion

IS events trigger a cascade of pathological changes, including neuronal degeneration, glial cell multiplication, and inflammation within the affected core area [18]. Neuronal cell death through apoptotic mechanisms following IS represents a major contributing factor to cerebral tissue damage [19]. The therapeutic approach to IS necessitates the preservation of neuronal viability and functionality within the penumbral region, particularly through the suppression of programmed cell death.

Research findings increasingly highlight the therapeutic value of naturally occurring substances as neuroprotectants, attributable to their complex composition, diverse molecular targets, and minimal adverse effects, which enable sustained administration [20,21]. Research data demonstrate the broad therapeutic potential of scutellarin across multiple disease states. The therapeutic applications of scutellarin encompass the enhancement of antioxidative mechanisms in hypoxic-ischemic cerebral damage, protection against hyperglycemia-induced testicular cell deterioration, and amelioration of coronary vessel function during cardiac ischemia-reperfusion events [22]. Furthermore, the anticancer activity of scutellarin has been demonstrated. Prior experimental work from our laboratory revealed scutellarin’s capacity to suppress neuroinflammatory processes through downregulation of Akt/NF-κB and p38 MAPK/JNK signaling cascades in LPS-stimulated BV2 microglia [23]. Additionally, scutellarin reduces apoptosis in PC12 cells mediated by BV2 microglial activation through the JAK2/STAT3 signaling pathway [9]. However, activation of this pathway alone does not fully account for scutellarin’s anti-apoptotic effects, suggesting the involvement of additional signaling pathways.

The emerging discipline of network pharmacology (NP) integrates multiple scientific domains, including systems biology, genomic analysis, and proteomic research. It predicts the interactions between compounds, active ingredients, and disease targets at a systemic level, establishing multi-layered networks (e.g., drug-component-target-disease) to identify potential key targets associated with drugs and diseases [24,25]. The computational technique of molecular docking employs various conformational analyses to assess molecular interactions between receptors and ligands, determining potential binding configurations and interaction strengths [26]. In this study, to identify potential target pathways through which scutellarin alleviates neuronal apoptosis after IS, network pharmacology analysis identified 1887 IS-related targets and 129 scutellarin potential therapeutic targets. Comparative bioinformatic analysis utilizing set theory methodology demonstrated 23 shared therapeutic targets between scutellarin intervention and ischemic stroke pathways. Subsequent evaluation of interconnected nodes in the PPI network architecture highlighted five crucial molecular mediators of therapeutic significance. CASP3, a pivotal executioner protease within the caspase family, orchestrates apoptotic cascade initiation through its interactions with both caspase-8 and caspase-9. The activation of CASP3 proceeds through two distinct mechanisms: an external receptor-dependent route and an internal mitochondrial-mediated cascade. Once activated, this protease catalyzes the degradation of multiple cellular components, including DNA structures, anti-apoptotic factors, and structural proteins, ultimately facilitating cellular death [27,28]. Moreover, inhibiting CASP3 has been shown to provide strong protection for neurons, preventing the death of ischemic and traumatic cells [29]. Upon activation, PTGS2, which maintains continuous expression in cerebral tissue [30], initiates inflammatory cascades. Scientific investigations reveal that PTGS2 enhances inflammatory mediator synthesis via simultaneous stimulation of PI3K/AKT and PKA/CREB pathways within neuronal cells, resulting in accelerated cell death and compromised neural development [31]. Studies extensively document the essential role of Epidermal Growth Factor Receptor in maintaining cellular viability, regulating cell cycle dynamics, and orchestrating differentiation mechanisms. Extensive oncological research has characterized EGFR’s fundamental role in cancer cell survival mechanisms. Contemporary neurological investigations have revealed marked EGFR expression reduction in compromised neural tissue, indicating its regulatory function in neuronal pathophysiology [32]. Research indicates that the role of EGFR in neuro injuries, such as IS, is dual. On the one hand, EGFR activation can inhibit apoptosis via the PI3K/AKT pathway, promoting cell survival and providing protection to damaged neurons. On the other hand, excessive or prolonged activation of EGFR may lead to abnormal cell proliferation or, through interactions with other pro-apoptotic pathways, promote neuronal apoptosis [33,34]. C-C motif chemokine ligand 2 (CCL2/MCP-1) belongs to the monocyte chemoattractant protein family and demonstrates ubiquitous expression throughout the neural environment, including glial cells, neurons, and cerebral microvascular endothelium. Through receptor-mediated signaling mechanisms, CCL2 functions as a key orchestrator of neuroinflammatory responses, facilitating the chemotactic migration of peripheral macrophages and endogenous microglial populations toward inflammatory foci within CNS tissues [35]. Moreover, the relationship between CCL2 and neuronal apoptosis has gained increasing attention. Studies suggest that CCL2 directly affects neuronal survival by activating the CCR2 receptor, promoting apoptosis, especially after IS, where the upregulation of CCL2 and accumulation of immune cells exacerbate neuronal death and brain tissue damage [36]. Caspase-1 (CASP1/ICE) undergoes activation via diverse inflammasome complexes during both subacute and recovery phases of ischemic stroke, resulting in amplified neuroinflammatory responses and exacerbated neuronal injury [37]. As a primary mediator of inflammatory cell death, CASP1 triggers pyroptotic mechanisms, which represent a unique pathway of cellular demise separate from conventional apoptotic processes. This cascade promotes the secretion of inflammatory mediators, notably the cytokines IL-1β and IL-18. This not only amplifies the local inflammatory response but also promotes apoptosis and necrosis [38]. Moreover, CASP1 activation demonstrates cross-talk with canonical apoptotic pathways, specifically through the facilitation of executioner caspase activation, such as CASP3, thereby potentiating cellular death mechanisms [39].

Our investigation employed molecular docking analysis to evaluate the interaction between scutellarin and essential target proteins. The computational results revealed spontaneous binding of scutellarin to all five crucial targets, with remarkable affinity scores. While molecular docking analysis demonstrates promising binding affinities between scutellarin and core targets, this static computational approach cannot fully capture the dynamic conformational changes occurring in physiological conditions. Thus, despite these encouraging in silico predictions, empirical validation remains essential to establish the biological significance of these molecular interactions. To understand the molecular basis of scutellarin’s neuroprotective effects, our research encompassed extensive bioinformatic analyses, including GO functional classification and KEGG pathway mapping, focusing on 23 shared targets between scutellarin and primary objectives. Analysis outcomes identified multiple critical signal transduction cascades, notably the PI3K-AKT signaling axis, HIF-1 mediated pathways, and TNF-related molecular mechanisms. Network pharmacology analysis, while providing valuable preliminary insights, is inherently constrained by the temporal and methodological limitations of existing databases. Despite its utility in establishing initial mechanistic frameworks, the computational predictions derived from this approach warrant thorough experimental validation to substantiate their biological relevance.

Based on the previous transcriptome sequencing results of MCAO rat brain tissues analyzed by our research group, we ultimately identified the PI3K/AKT signaling pathway as one of the potential target pathways through which scutellarin alleviates neuronal apoptosis after ischemic stroke (IS). This pathway will be further validated in subsequent experiments. The PI3K/AKT signaling cascade functions as a fundamental survival pathway, demonstrating robust cell death prevention capabilities [40]. This fundamental signaling cascade orchestrates diverse cellular processes, including development, differentiation, survival, protein synthesis, and metabolic regulation in mammalian systems. Activated PI3K phosphorylates AKT, which in turn regulates downstream substrates, triggering a cascade of reactions that influence apoptosis and cell cycle regulation [41,42]. The PI3K/AKT signaling cascade serves as a critical orchestrator of molecular mechanisms in cerebral ischemia, playing an essential role in brain homeostatic regulation. Impaired functioning of this signaling cascade has been linked to multiple pathological conditions, including metabolic disorders, neural degeneration, cerebrovascular events, and malignancies [43]. Accumulating evidence demonstrates the pivotal role of PI3K/AKT signaling in modulating oxidative stress responses and inflammatory cascades during ischemic injury [44]. Experimental stroke research frequently employs the rodent MCAO as a standardized and validated paradigm to replicate human ischemic conditions [45]. In this study, experiments using the MCAO rat model showed that 3 days post-MCAO, the ischemic cortex exhibited significant neuronal apoptosis, reduced Nissl bodies, and poorly defined cell contours. After scutellarin intervention, the number of apoptotic neurons decreased significantly, and neuronal morphology showed signs of recovery. The therapeutic application of scutellarin promoted PI3K/AKT signal transduction via enhanced phosphorylation and elevated Bcl-2 protein levels, while simultaneously diminishing the expression of apoptotic regulators Bax and activated caspase-3. Application of the PI3K antagonist LY294002 diminished scutellarin’s therapeutic efficacy, as evidenced by reduced phosphorylation of PI3K and AKT, and decreased Bcl-2 levels, accompanied by heightened expression of cell death markers, including Bax and activated caspase-3. These experimental observations establish that scutellarin’s neuroprotective properties operate via the enhancement of PI3K/AKT signaling, resulting in the suppression of neuronal death pathways.

Extensive laboratory studies, encompassing both cellular and animal models, reveal scutellarin’s protective effects against ischemia-induced neural death, primarily via PI3K/AKT pathway stimulation. These findings establish a mechanistic foundation for the therapeutic application of scutellarin in IS and related ischemic-hypoxic cardiovascular disorders. Despite scutellarin’s potent neuroprotective properties, its therapeutic efficacy is constrained by limited oral bioavailability. Recent research indicates that plant-derived compounds may augment their therapeutic effects through intestinal microbiome interactions, with tea polyphenols demonstrating enhanced metabolic and anti-inflammatory benefits via microbiota-dependent SCFA generation [46]. Similarly, ferulic acid (FA), another natural phenolic compound, has been found to modulate gut microbiota composition, increase SCFA production, and alleviate intestinal inflammation, thus improving conditions such as obesity and metabolic diseases [47]. Therefore, future studies could explore whether scutellarin exerts similar effects by modulating gut microbiota, potentially improving the pathological features of neurodegenerative diseases. Further investigation into scutellarin’s role in the gut microbiota-immune-neuro axis could provide stronger scientific support for its clinical application in neurological disorders.

## 4. Materials and Methods

### 4.1. Data Sources

The GSE97537 dataset matrix file was downloaded from the GEO database (https://www.ncbi.nlm.nih.gov/geo/) using “ischemic stroke” as the search term. Expression profiling was conducted using the GPL1355 (Affymetrix Rat Genome 230 2.0) platform, analyzing samples from 7 MCAO-induced and 5 sham-operated rodents.

### 4.2. Screening and Visualization of Differentially Expressed Genes

The GSE97537 dataset was read using the GEOquery 2.72.0 package, and probes corresponding to multiple molecules were removed. Differential gene expression analysis employed the limma 3.60.4 R package, with significance criteria set at fold change > 1.5 and *p*-value < 0.05. Visualization of differential expression patterns utilized ggplot2 3.5.1 for volcano plot generation and pheatmap for hierarchical clustering representation.

### 4.3. Screening of Potential Scutellarin Targets and Venn Diagram Construction

The SMILES format file for scutellarin was obtained from PubChem (https://pubchem.ncbi.nlm.nih.gov). Target prediction analysis was conducted using dual computational platforms: SwissTargetPrediction (http://swisstargetprediction.ch) and PharmMapper (http://lilab-ecust.cn/pharmmapper/index.html), utilizing the scutellarin structural data. Protein nomenclature underwent standardization through UniProt database (https://www.uniprot.org) reconciliation, followed by elimination of redundant entries. The Venn online tool (https://bioinfogp.cnb.csic.es/tools/venny) was used to identify the intersection of scutellarin targets and the DEGs from the GSE97537 dataset, yielding common potential targets.

### 4.4. Protein–Protein Interaction (PPI) Network Construction and Core Target Screening

The overlapping targets were imported into the STRING database (https://string-db.org/) with the species set to “Homo sapiens” to construct the PPI network. Network visualization and analysis were executed through Cytoscape 3.7.1 platform implementation. Topological network analysis was conducted utilizing the CytoHubba 3.7.1 plugin to evaluate interaction parameters. Network connectivity was quantified through node degree values, where higher degrees indicate greater nodal significance within the interaction network. The top 5 hub genes were identified based on their degree values.

### 4.5. GO Functional and KEGG Pathway Enrichment Analysis

The overlapping targets were subjected to GO functional annotation and KEGG pathway enrichment analysis using the Metascape database (http://metascape.org/gp/index.html) with the species set to “Homo sapiens”. GO analysis examined three functional categories: biological processes (BP), molecular functions (MF), and cellular components (CC). Pathway enrichment significance was established at a threshold of *p* < 0.05 for KEGG analysis. Enrichment analysis outcomes were graphically represented using specialized bioinformatics visualization tools (http://www.bioinformatics.com.cn/).

### 4.6. Molecular Docking

The 3D structures of the top 5 core targets were obtained from the PDB database (http://www.rcsb.org/) in “pdb” format. Using PyMOL 2.4.0 software, water molecules and irrelevant ligands were removed from the proteins. The macromolecular proteins were prepared for docking by adding hydrogen atoms and calculating charges with AutoDock Vina 1.5.6. The optimized protein structures were converted to PDBQT format for receptor modeling. Computational docking simulations were executed via the AutoDock Vina platform, followed by structural visualization analysis. Optimal molecular conformations were selected based on minimum binding energy parameters.

### 4.7. Experimental Animals and Grouping

Fifteen male Sprague-Dawley rats (200–250 g) were obtained and housed under SPF conditions from Kunming Medical University’s Laboratory Animal Center. The animals were maintained in a controlled environment with standardized temperature, circadian rhythm (12 h light/dark cycles), and ad libitum access to nutrition at the Basic Medical College’s animal facility. Following a seven-day adaptation period, ischemic stroke was induced through the middle cerebral artery occlusion procedure. The animals were randomized into three experimental groups: sham operation (sham), MCAO model (MCAO), and scutellarin treatment (MCAO + S). Scutellarin (100 mg·kg^−1^) [23] or saline was administered intraperitoneally 2 h before MCAO and at 12, 24, 36, 48, and 60 h post-surgery. All the rats were anesthetized and sacrificed 3 days after surgery for tissue collection.

### 4.8. Drugs and Reagents Consumables

Scutellarin: Ronghe Medical Technology Co., Ltd. (20 mg; molecular formula: C_21_H_18_O_12_; purity: ≥98%, Shanghai, China). Cell culture six-well plates and culture flasks: Lanjieke Technology Co., Ltd. (Beijing, China). F12/DMEM (1:1) high-glucose medium, glucose-free medium, and fetal bovine serum: Thermo Fisher Scientific (Waltham, MA, USA). Penicillin/streptomycin solution, RIPA lysis buffer, and DAPI-containing fluorescent mounting medium: Solarbio Science & Technology Co., Ltd. (Beijing, China). Nissl staining reagent and protease inhibitors: Beyotime Biotechnology Co., Ltd. (Beijing, China). Phosphatase inhibitors: MCE Co., Ltd. (Shanghai, China). BCA protein assay kit: Dalian Meilun Biotechnology Co., Ltd. (Dalian, China). Rabbit anti-p-PI3K: Bioss Biotechnology Co., Ltd. (Beijing, China). Apoptosis detection kit, primary antibodies against signaling proteins, and fluorophore-conjugated secondary antibodies:Proteintech (Wuhan, China). Rabbit anti-Bax and Bcl-2 antibodies: Shenyang Wanlei Biotechnology Co., Ltd. (Shengyang, China). PI3K inhibitor LY294002: MCE Co., Ltd. (Shanghai, China). Total RNA extraction kit with spin columns: Tiangen Biotech Co., Ltd. (Beijing, China). Reverse transcription and amplification kits: Nanjing Vazyme Biotech Co., Ltd. (Nanjing, China).

### 4.9. Cell Culture

PC12 cells were obtained from Prof. Ling Eng-Ang at the National University of Singapore. Cryopreserved PC12 cells were recovered from −80 °C storage and thawed at 37 °C with gentle agitation until homogeneous. The cell suspension was transferred to F12/DMEM (1:1) high-glucose medium, supplemented with 10% FBS and 1% penicillin/streptomycin. The cells were centrifuged at 1000 rpm for 5 min, then the supernatant was discarded, and fresh medium was added. After thorough mixing, the cells were transferred into a cell culture flask, gently shaken, and incubated in a 37 °C, 5% CO_2_ incubator. Cells were monitored daily and passaged minimally twice prior to experimental use.

### 4.10. Cell Grouping

PC12 cells were maintained and seeded in multi-well culture plates. The cells were divided into four groups: control group (Control), oxygen-glucose deprivation group (OGD), oxygen-glucose deprivation + scutellarin group (OGD + S), and oxygen-glucose deprivation + scutellarin + PI3K inhibitor LY294002 group (OGD + S + I). For the PI3K inhibition group, LY294002 was pre-administered 1 h before scutellarin treatment, followed by scutellarin incubation for 1 h. Scutellarin was administered at 0.54 μM, as optimized in previous studies. OGD conditions were established using glucose-deprivation and hypoxic environment (95% N_2_, 5% CO_2_). The OGD model was established by subsequent incubation at 37 °C for 4 h.

### 4.11. Detection of Apoptosis and Nissl Staining

Programmed cell death evaluation was performed utilizing the TUNEL methodology following standard protocols. TUNEL-positive cells were visualized by fluorescence microscopy and quantified using ImageJ 3.0 analysis (apoptotic cells appear green). Neuronal morphology was assessed via toluidine blue-based Nissl staining procedures according to standardized protocols. Microscopic examination and digital documentation of Nissl-positive structures were performed for subsequent analysis.

### 4.12. Western Blot Analysis

Cortical specimens were extracted under anesthesia and processed through ultrasonication in lysis buffer under temperature-controlled conditions. Sample fractionation was achieved through high-speed centrifugation (14,000 r·min^−1^, 4 °C, 10 min), with subsequent collection of the supernatant fraction. Protein concentrations were determined by BCA assay. Denatured protein samples underwent SDS-PAGE separation. Separated proteins were electroblotted onto PVDF membranes. Membranes were blocked in 5% non-fat milk for 2 h at room temperature. Primary antibody incubation was performed overnight at 4 °C after TBST washing. Following TBST washes, membranes were probed with secondary antibodies for 1h at room temperature. Immunoreactive bands were visualized using ECL detection system. Densitometric analysis of protein expression was performed using ImageJ quantification tools.

### 4.13. Immunofluorescence Staining

Cryosectioned brain tissue specimens underwent thermal equilibration (30 min, ambient temperature) followed by phosphate-buffered saline rinses. Endogenous peroxidase activity was quenched prior to PBS washing and subsequent blocking with 10% caprine serum (1 h, room temperature). Tissue sections underwent overnight immunoreaction with specific primary antibodies under controlled humidity conditions at 4 °C. Following PBS washing cycles, immunodetection proceeded with fluorophore-conjugated secondary antibodies (AF488 or Cy3-labeled anti-rabbit IgG) under light-protected conditions (1 h, room temperature). Nuclear counterstaining and section mounting were accomplished using DAPI-supplemented fluorescence preservation medium. Quantitative fluorescence microscopy was performed within a seven-day window, with signal intensity analysis conducted via ImageJ densitometry.

### 4.14. Real-Time Reverse Transcription Polymerase Chain Reaction (RT-PCR)

RNA was isolated from PC12 cells and quantified spectrophotometrically. Reverse transcription was performed according to manufacturer’s specifications. The cDNA was then used as a template for the PCR steps. Reaction mixtures were prepared following the kit’s protocol for RT-PCR experiments. The mRNA expression levels of pathway-enriched genes were detected, with Actin serving as the internal control to determine the relative mRNA levels. Relative expression was calculated using the 2^−△△Ct^ method; primer sequences are listed in Table 3.

### 4.15. Statistical Analysis

Intergroup variations were evaluated through one-way analysis of variance methodology. Statistical analyses were performed using GraphPad Prism 8.0. Experimental outcomes were presented as arithmetic means accompanied by standard deviations ($\bar{x}$ ± s). Statistical significance was established at a probability threshold of 0.05 (*p* < 0.05).

## Figures and Tables

**Table 1:** Top five core gene target proteins ranked by degree value.

Gene Name	Protein Name	Betweenness	Closeness	Degree
CASP3	Caspase-3	0.1774088	0.73076923	13
PTGS2	Prostaglandin G/H synthase 2	0.19056669	0.7037037	12
EGFR	Epidermal growth factor receptor	0.16846979	0.7037037	12
CCL2	C-C motif chemokine 2	0.03793512	0.67857143	11
CASP1	Caspase-1	0.09090783	0.65517241	10

Betweenness: Measures the node’s role as a “bridge”, indicating the number of shortest paths passing through the node. Closeness: Represents the average shortest path length from the node to all other nodes; higher values indicate faster information spread. Degree: The number of direct connections a node has; higher values indicate stronger connectivity.

**Table 2:** Molecular docking of scutellarin with core target proteins.

Target Name	PDB	Binding Energy (kcal/mol)	Amino Acid Residues
CASP1	3E4C	−12.7	LEU-258,GLN-257,GLU-241,ARG-391,THR-388,MET-386,SER-333,SER-298,PRO-290
CASP3	1CP3	−7.9	TYR-197
CCL2	1DOK	−7.4	ASN-14,THR-32,THR-10,ARG-29
EGFR	1IVO	−9.0	ARG-84,ASN-86,ARG-228,ASN-247
PTGS2	5F19	−9.9	SER-49,ASN-34,TRP-323,GLN-327,ASP-325,GLU-326

**Table 3:** Primer sequence for RT-PCR.

Gene	Forward Primer (5′–3′)	Reverse Primer (5′–3′)
EGFR	AGGAGGTGGCTGGCTATGTTCT	AGGACGGCTAAGGCGTAGGT
EIF4EBP1	CCCAAAGGACCTGCCAACCATT	CACCACCTGCCCGCTTATCTTC
FGF2	AAGAGCGACCCACACGTCAAAC	CAGCAGCCGTCCATCTTCCTTC
β-Actin	TGCTATGTTGCCCTAGACTTCG	GTTGGCATAGAGGTCTTTACGG

## Data Availability

The data are contained within the article.
